# Management of Type 1 Diabetes in a school setting: effectiveness of an online training program for school staff

**DOI:** 10.3389/fpubh.2023.1228975

**Published:** 2024-01-04

**Authors:** Marta Bassi, Marta Scalas, Giordano Spacco, Viola Perasso, Daniele Franzone, Marina Francesca Strati, Francesca Dufour, Barbara Lionetti, Francesca Rizza, Stefano Parodi, Giuseppe d’Annunzio, Nicola Minuto

**Affiliations:** ^1^Pediatric Clinic and Endocrinology Unit, IRCCS Istituto Giannina Gaslini, Genoa, Italy; ^2^Department of Neuroscience, Rehabilitation, Ophthalmology, Genetics, Maternal and Child Health, University of Genoa, Genoa, Italy; ^3^Epidemiology and Biostatistics Unit, Scientific Directorate, IRCCS Istituto Giannina Gaslini, Genoa, Italy

**Keywords:** Type 1 Diabetes, education, school, teachers, telemedicine, COVID-19

## Abstract

**Background and aims:**

Since Type 1 Diabetes (T1D) onset usually occurs at a young age, a relevant number of affected people attend school for most of their time; it is necessary that school personnel receive appropriate education and training. We aimed to evaluate the effectiveness of the online training program offered by IRCCS Istituto Giannina Gaslini during and after COVID-19 pandemic.

**Methods:**

The Institute’s Diabetes team offered an online training program to school staff of the Region during COVID-19 pandemic. A validated questionnaire was proposed to all the schools in which training meetings were held in the previous 2 years (2020–2021 and 2021–2022). The questionnaire consisted of four sections: Section 1 (Socio-demographical data), Section 2 (Theoretical knowledge on T1D), Section 3 (Confidence in handling T1D), and Section 4 (Practical skills and Glucagon Administration). To evaluate the effectiveness of the online training program, the answers between participants (Group A) and non-participants (Group B) were then compared.

**Results:**

225 subjects from 19 schools participated in the survey. People who participated to the training (Group A, *n* = 53) demonstrated better T1D theoretical knowledge compared to non-participants (Group B, *n* = 154; *p* < 0.001). Group A revealed to feel more confident in the management of children with T1D during scholastic (*p* = 0.006) and extra-scholastic activities (*p* = 0.01), in supporting the children in the administration of insulin (*p* < 0.001) and in recognizing hypoglycaemia (*p* = 0.006). Moreover, results confirmed good levels of confidence among scholastic personnel who participated in the training of administration of glucagon in case of severe hypoglycaemia.

**Conclusion:**

School staff who took part in the online training program on management of T1D showed better theoretical knowledge and better confidence in the management of daily needs and possible emergencies of students with T1D. It appears essential to offer educational programs on T1D for school staff by implementing the use of technological tools to reach a wider population. Moreover, it is advisable to offer a more practical approach, involving educational nurses.

## 1 Introduction

Type 1 Diabetes (T1D) is the most common form of diabetes in children and adolescents (1). The number of young people with diabetes attending school is increasing, therefore the importance of support and management of the child is growing not only among families and the health care system, but also among school staff. The International Society for Pediatric and Adolescent Diabetes (ISPAD) underlined that a collaborative approach among parents, the student’s health care team and schools, together with advancements in communication and technology, should be used to optimally support students for successful diabetes management at school (2).

The correct management of T1D requires blood sugar monitoring and insulin replacement therapy and has completely changed in the last years. Continuous Glucose Monitoring (CGM) and Advanced Hybrid Closed Loop (AHCL) systems allow a simpler management of the disease, thanks to the continuous glucose monitoring and hypoglycaemia alarms, and to the decreasing need for intervention required by the caregiver and the patient for therapeutic decisions (3–5).

Children and adolescents with T1D spend most of their day in school and must be allowed to monitor their glucose levels, administer insulin, and to treat hypo- or hyperglycaemia at any time during school, with adult supervision and assistance if needed. All school personnel must receive appropriate diabetes education and the training is the healthcare team and family’s responsibility. The content of the training is defined by the health care team and should include basic understanding of diabetes and of the emergency management of hypoglycaemia (2). Despite clear international recommendations, unfortunately school staff’s knowledge about diabetes and its management is often insufficient in many countries (6–11) and this situation raises concerns and feelings of fear and stress in school staff who approach the child or adolescent with T1D (12). Families and school staff seem to be aware of their lack of knowledge and concerns and very interested in promoting training and support initiatives for schools (12–15). Another aspect that clearly emerges from literature on this topic is the need for local and national institutions to provide a clear and specific legislation for the management of T1D within schools, in order to ensure standardized training and school nurses and to avoid gaps between local school practices that lead to possible inequity of care (16–19). The need to implement educational programs aimed at school staff to create a safe and relaxed environment where the child/adolescent with diabetes can spend his or her daily life emerges from all the mentioned studies.

Some studies have evaluated the effectiveness of educational interventions aimed at school staff in terms of knowledge about diabetes and level of confidence in its management. Bechara et al. and Gocke et al. evaluated the effectiveness of two different educational interventions based on providing online free materials: the DPS (“Diabetes Program at School”) in Turkey and the KiDS Project in Brazil (Kids and Diabetes in School project supported by ISPAD and IDF-International Diabetes Federation). Both programs proved to be effective in improving knowledge and attitude of school staff in the management of diabetes at school (20, 21). Dixe et al. (22) showed the effectiveness of a 6-h structured educational program performed by previously trained nurses in Portuguese schools in enhancing knowledge and confidence while supporting students. Gurunathan et al. (23) evaluated a 6-h pediatric endocrinologist-led structured educational program showing the significant impact of the intervention in reducing fear of hypoglycaemia in school staff. Finally, Tournilhac et al. (24) demonstrated the effectiveness of a video training program in improving the confidence level of teachers in the administration of intramuscular glucagon.

In Italy, there are no specific regulations regarding the education of school staff for the management of T1D, but there are strategic guidelines created by national associations in collaboration with the Ministry of Health (25). Differently from other countries, there is no school nurse role, however dedicated nurse figures can be assigned to a T1D student to carry out interventions during school hours, especially for younger children who attend kindergarten and primary schools who are not able to independently perform complicated actions such as dealing with the pump and the sensor, insulin administration, or carb counting. Since the figure of the school nurse is not guaranteed and the dedicated nurse is not present for the entire school day, it is essential that school personnel are provided with the basic knowledge to support the child in case of hypoglycaemia (event that can occur unpredictably at any time of the day). The training program offered to schools by the IRCCS Istituto Giannina Gaslini (IGG) Regional Center for Pediatric Diabetology was created by the collaboration between the hospital and the Youth Diabetes Regional Association (ADG Liguria—Associazione Diabete Giovanile Liguria). The educational intervention consisted of a theoretical and practical meeting between health care professionals and school staff performed in the Institutes (kindergartens, primary and secondary schools) attended by patients followed at the Center. Until 2020, the meetings were always held in the presence of the healthcare professionals in school facilities, but since the COVID-19 pandemic, to avoid interruption of the service, given its importance, online meetings started taking place. During the pandemic, the use of telemedicine in pediatric diabetology was started and implemented, making it possible to maintain a correct follow-up in T1D children and adolescents during this difficult period (26–29). Telemedicine and telenursing have been proven to be effective in supporting T1D patients and to be associated with a high degree of patient and family satisfaction (30–35). Therefore, telemedicine continues to be used in pediatric patients with T1D with different organizational and care models (36–38). Telemedicine and telenursing continue to be used today in our center where it can be useful and beneficial for the patient. Likewise, the online educational meetings with schools continue to be carried out in case of particular needs (i.e., very distant schools, temporary educational intervention on more than one institution) or simply to make it possible for several schools to participate at the same time.

The aim of this study was to evaluate the effectiveness of the online school staff training program conducted by healthcare professionals of IRCCS IGG Regional Center for Pediatric Diabetology. The evaluation of the effectiveness of the online training program constitutes an important starting point for the possible continuation of the program even outside the pandemic period. The study project also included the validation of a questionnaire, as in previous studies many aspects (theoretical knowledge, self-confidence, and practical skills) had been addressed separately and in this study we wanted to analyze them all together.

## 2 Materials and methods

### 2.1 Study design

The aim of the study was to evaluate the effectiveness of the online school staff training programs conducted by healthcare professionals comparing the level of theoretical knowledge and confidence in the management of T1D among school personnel who had attended the training (Group A) and who had not attended (Group B).

The structured school staff training program conducted by healthcare professionals of IRCCS IGG Regional Center for Pediatric Diabetology consists of a theoretical part (conducted by the diabetologist), a practical part (conducted by the nurse) and a final part dedicated to the patient or specific questions by the school staff.

- The theoretical program is planned to cover basic knowledge about T1D (definition of T1D and difference with T2D, symptoms of T1D onset), management of hypoglycaemia and hyperglycaemia, management of physical activity and extracurricular activities and basic principles and management of insulin therapy (differences between basal insulin and rapid-acting insulin, basic meal bolus management, importance of the waiting times between the insulin injection, and the start of the meal).
- The structured practical program includes education on skills such as: basic use of glucometers and continuous glucose monitoring, and use of insulin pumps. An essential aspect of the practical education is the management of severe hypoglycaemia with loss of consciousness and the procedure for administering intramuscular or nasal glucagon is always taught. Due to the COVID-19 pandemic, the meetings during the 2020–2021 and 2021–2022 school years were held telematically, therefore the practical part was limited because the school staff did not have the opportunity to try the techniques but only to see them done by the nurse.

The study was conducted between June and October 2022 and consisted of two different phases: the creation and validation of the questionnaire and its administration to the school staff.

### 2.2 Validation of the questionnaire

A new questionnaire was created based on three validated questionnaires already used in previous studies and used to assess general knowledge about diabetes, self-confidence, and management skills of diabetes-related problems of teachers and school staff (6, 7, 39). These questionnaires have been merged to create a new evaluation tool that has been validated by a group of six experts in the field of diabetes working at IRCCS IGG (a pediatric diabetologist, a resident in pediatrics, a psychologist, and three pediatric nurses). Each component of the group was asked to rate from 1 to 4 the relevance and comprehensibility of each item (40).

The validated questionnaire consisted of four sections (Supplementary Table 1):

- *Section 1—socio-demographical characteristics of participants* (i.e., age, years of working experience in school, education level, role at school, and personal or family history of T1D)
- *Section 2—level of theoretical knowledge about T1D* (i.e., etiology, clinical presentation, main acute complications, and therapy among others). The response mode for this section was multiple-choice.
- *Section 3—level of self-confidence in handling T1D* related daily needs and emergencies, both at school and in extra-school settings. In this section, responses were given on a five-point Likert scale ranging either from “excellent” to “poor” or from “agree completely” to “do not agree at all.”
- *Section 4—practical skills and Knowledge and confidence in the methods of administering glucagon* in case of severe hypoglycaemic events. Since this study involves participants that have followed trainings both in intramuscular and nasal administration, the questions in this part were related to both methods of administration alternately. In this section, responses were given either on a five-point-Likert scale ranging from “agree completely” to “do not agree at all” or a four-point Likert scale ranging from “not problematic” at all to “very problematic.”

The first and the second section of the questionnaire were completed by all participants. The third section was filled out only by participants that were working or that had worked in classes with a student with T1D, while the fourth section was filled out only by participants that had attended the training.

### 2.3 Study population

The questionnaire was administered online and anonymously to school staff members that agreed to participate in the study. Participation in the study was offered to all schools where the training was carried out by healthcare professionals of IRCCS IGG Regional Center for Pediatric Diabetology in the years 2020–2021 and 2021–2022 through a proposal for participation and explanation of the aims of the study to each headmaster. After the proposal, the link to access the anonymous questionnaire was sent to the headmasters of the participating school, who provided it to all school employees. Over the 2-year period, 68 schools and 795 teachers took part in the online training program, corresponding to a variable number of approximately 10–12 training participants for each school. In the 19 schools that took part in the study, the estimated number of training participants was approximately of 190–220 teachers. All members of school staff (teachers, support teachers, head teachers, administrators, auxiliary staff, and social educators) working in kindergartens, in primary and secondary schools could complete the questionnaire, regardless of individual participation in the training or direct contact with students affected by T1D. Considering the average size of the schools in Liguria, for each school participating in the study, the questionnaire was probably proposed to approximately 40–60 teachers. Participants affected by T1D or with family members affected by T1D were excluded from the study, as they already possessed good knowledge and management skills about diabetes despite the training. The responses to the questionnaires were collected via an anonymous online form associated with the questionnaire link.

### 2.4 Statistical analysis

Content validation of the questionnaire was assessed calculating the Content Validity Index for each item (I-CVI) and for the whole questionnaire (“scale validity index,” S-CVI) (40). According to the Polit and Beck suggestions, an item was considered as validated if it was assigned an I-CVI > 83% for both the relevance and the comprehensibility, while the corresponding cut-offs for S-CVI were set at 90% (40).

The validated questionnaire was analyzed using absolute frequencies and percentages to summarize qualitative variables. The comparison between groups was performed by the Pearson chi square test or the Fisher exact test when appropriate. To increase statistical power, we grouped grades 3, 4, and 5 of Likert scale in a single grade. The number of correct answers was resumed by the median value and the related interquartile range (IQR); the Mann–Whitney U test was applied for group comparisons. All analyses were carried out using the software STATA for Windows, version 13.1 (Stata Corporation, College Station, Texas, United States).

## 3 Results

### 3.1 Validation of the questionnaire

At the end of the first round of validation, each item of the questionnaire reached a content validity index (I-CVI) of 100%, for both the relevance and the comprehensibility, except item 8 that received a negative rate by one validator for relevance (I-CVI = 83.3%), still passing the validation. Accordingly, S-CVI was 100% for the comprehensibility and 98% for the relevance.

### 3.2 Survey

The collected data included a total of 225 questionnaires completed by participants from 19 schools of Liguria. Most of the participants were older than 40 years (82%) and had more than 10 years of work experience in school (39%). Participants were distributed quite evenly among different grades of school (13% from kindergartens, 30% from primary schools, 27% from first grade secondary schools, and 30% from second grade secondary schools) and most of the participating schools were in the province of Genoa (88%). Almost all participants were teachers (80% were class teachers and 19% support teachers). Socio-demographic and work profile characteristics of this population are shown in Supplementary Table 2. 18 participants did not complete the subsequent sections of the questionnaire because they had a family member with T1D. Accordingly, statistical analyses of the Section 2 of the questionnaire (*Level of theoretical knowledge about T1D*) were carried out on 207 participants. The socio-demographic and work profile characteristics of the study population according to the two groups (participants and non-participants in the program) are shown in Table 1. No significant difference was found between the two groups, except for school level, which showed a lower proportion of participants in the online program who worked in a high school (9.4 vs. 38.3%, *p* < 0.001).

**Table 1:** Socio-demographic and work profile characteristics of the study population by group.

	Participants to the online training program				
Patient characteristics	No (*n* = 154)		Yes (*n* = 53)		
	*n*	%	*n*	%	*p*
Age group					*0.159*
< 30 years	7	4.6	1	1.9	
30–39 years	26	16.9	4	7.6	
≥ 40 years	121	78.6	48	90.6	
Duration of work experience in school					*0.106*
< 5 years	29	18.8	3	5.7	
5–9 years	26	16.9	8	15.1	
10–19 years	45	29.2	17	32.1	
≥ 20 years	54	35.1	25	47.2	
Education					*0.545*
Professional school diploma	5	3.3	0	0.0	
High school diploma	30	19.5	14	26.4	
Graduation	89	57.8	29	54.7	
Postgraduate higher education	30	19.5	10	18.9	
Professional role					*0.173*
Teacher	123	79.9	42	79.3	
Support teacher	29	18.8	9	17.0	
Other staff members	2	1.3	0	0.0	
Headmaster	0	0.0	2	3.8	
School level					*< 0.001*
Kindergarten	16	10.4	11	20.8	
Primary school	45	29.2	15	28.3	
Middle school	34	22.1	22	41.5	
High school	59	38.3	5	9.4	
Teaching subject					*0.723*
Science subjects	18	11.8	7	13.7	
Other subjects	134	88.2	44	86.3	
Province of school					*0.033*
Genova	134	87.0	45	84.9	
Savona	1	0.65	0	0.0	
La Spezia	0	0.0	3	5.7	
Imperia	19	12.3	5	9.4	

**Figure 1:**
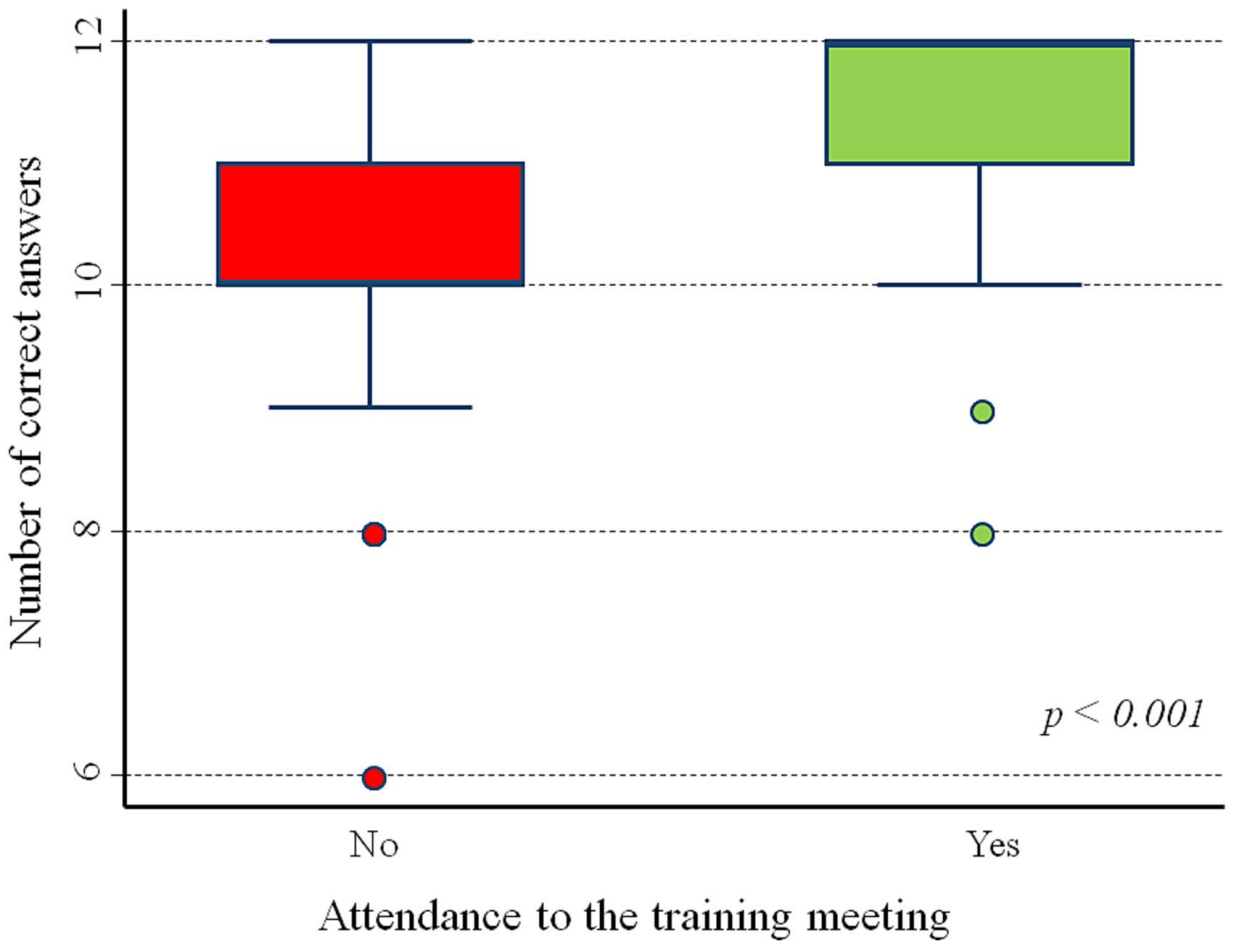
Section 2: Global difference in theoretical knowledge about T1D between Group A and Group B.

**Figure 2:**
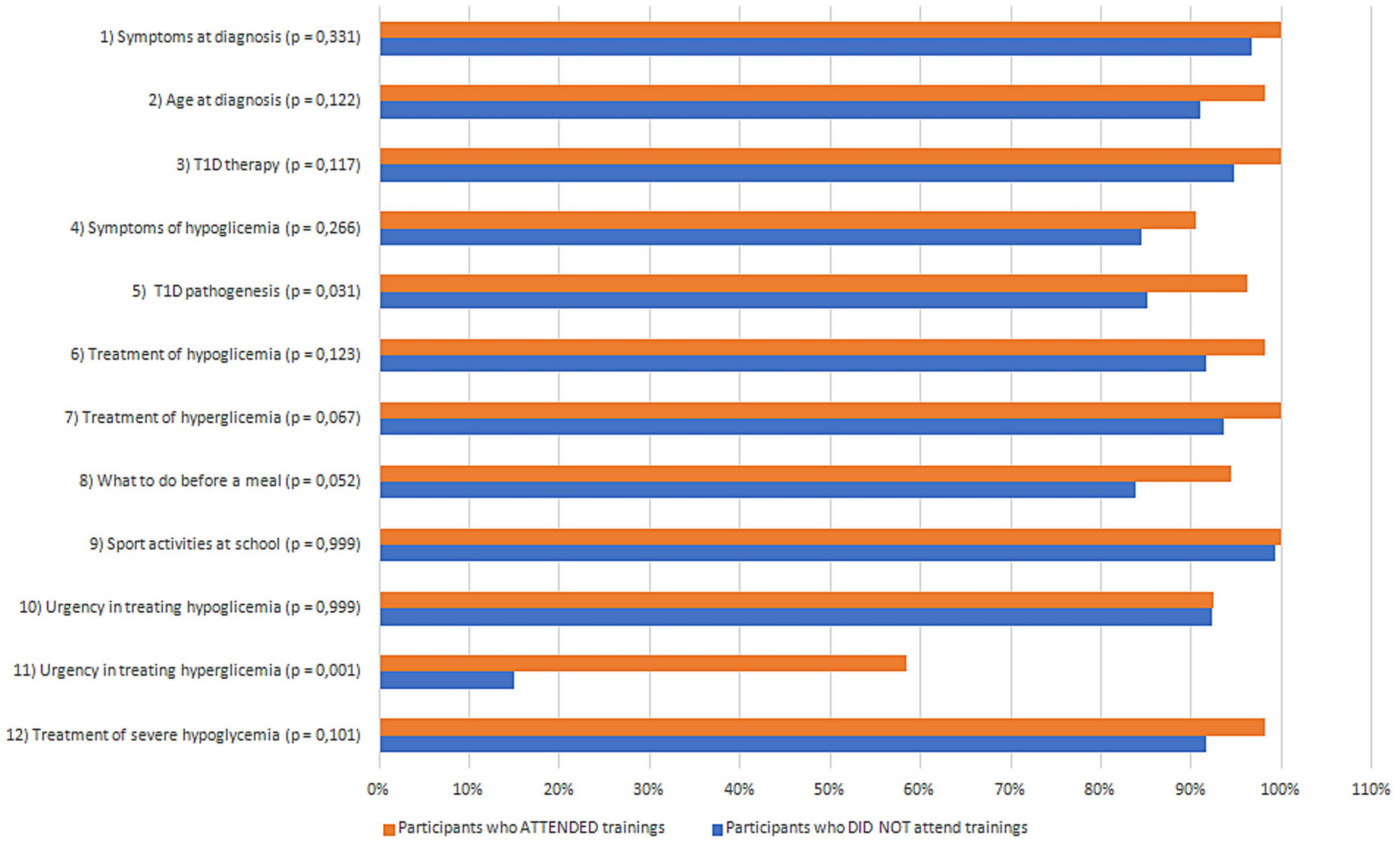
Section 2: Differences in theoretical knowledge about T1D for single item between Group A and Group B.

Overall, data showed a significant difference in theoretical knowledge about T1D (measured as the number of correct answers) between 53 participants that attended the training (Group A: median 10, IQR: 10–11) and 154 participants who did not (Group B: median 12, IQR: 11–12, *p* < 0.001; Figure 1). The comparison of the answers for single item between the two groups is shown in Figure 2 and Supplementary Table 3. The analysis of the single items showed a statistically significant higher frequency of correct answers in group A than in Group B for the questions related to the following aspects: etiopathogenetic mechanism of T1D (96.2 vs. 85.1%, *p* = 0.031) and management of hyperglycaemia (58.5 vs. 14.9%, *p* < 0.001). A difference approaching the significance was also found for the question relating to the management of the pre-meal insulin bolus (94.3 vs. 83.8%, *p* = 0.052).

Section 3 of the questionnaire (*Level of self-confidence in handling T1D*) was filled out only by the 82 participants that were working or that had worked in classes with a student with T1D. Among them, 53 attended the training (Group A) and 29 did not (Group B). The answers showed significant differences between the two groups in terms of perception of: theoretical knowledge (good/very good: 49.1% in Group A vs. 34.5% in Group B, *p* = 0.04), ability in managing T1D (52.9 vs. 27.6%, *p* = 0.004), confidence in managing T1D (68.0 vs. 24.1%, *p* < 0.001), recognition of symptoms of hypoglycaemia (58.5 vs. 24.1%, *p* = 0.006), attention to the prompt availability of sugar to treat hypoglycaemia (Agree: 88.7 vs. 51.7%, *p* < 0.001), ability to support the student in managing insulin therapy (67.9 vs. 27.6%, *p* < 0.001), and safety in managing the student in extra-curricular activities and school trips (73.6 vs. 44.8%, *p* = 0.010). No differences were found between the two groups in terms of confidence in direct contact with parents and health professionals for student management. Finally, the global evaluation of the teaching experience with a T1D student showed significant differences between those who participated in the training and those who did not (*p* = 0.001). Results of Section 3 of the survey are shown in Table 2.

**Table 2:** Differences in level of self-confidence in handling T1D between Group A and Group B.

	Participants to the online training program				
*Level of self-confidence in handling T1D*	Yes (*n* = 53)		No (*n* = 29)		
	*n*	%	*n*	%	*p*
Assessment of T1D knowledge					
Good–Very good	26	49.1	10	34.5	*0.040*
Assessment of T1D management skills					
Good–Very good	28	52.9	8	27.6	*0.004*
Recognition of hypoglycemia					
Good–Very good	31	58.5	7	24.1	*0.006*
Confidence in managing T1D student					
Agree	36	68.0	7	24.1	*<0.001*
Check for sugar availability in classroom					
Agree	47	88.7	15	51.7	*<0.001*
Confidence in managing T1D student in all school activities					
Agree	39	73.6	13	44.8	*0.010*
Confidence in support T1D student during insulin administration					
Agree	36	67.9	8	27.6	*<0.001*
					
Agree	48	90.6	27	93.1	*0.999*
Effective communication with healthcare professionals					
Agree	46	86.8	22	75.9	*0.209*
TD1 student teacher experience					
Very good–Good	52	98.1	21	72.4	*0.001*

Section 4 of the questionnaire *(Practical skills and Knowledge and confidence in the methods of administering glucagon)* was filled out only by the 53 participants that had attended the training. Most children (56.6%) self-monitor glucose levels without teacher supervision, while less than half of T1D students (39.6%) self-manage episodes of hypoglycaemia without teacher help and supervision. Regarding the support in the administration of insulin, 24.5% of the T1D students administered insulin themselves, 28% of them are supervised by the teacher and in the majority of cases (47.2%) they are supervised by other people. Among the 53 participants, 40 were trained to use intramuscular glucagon, 4 were trained to use nasal glucagon, and 5 were trained to use both intramuscular and nasal glucagon; of all of them, 4 did not respond to section 4 of the questionnaire. A global good self-confidence in administration can be noticed regardless of the type of formulation (Tables 3, 4). It was not possible to make a comparison between the two methods of administration due to the small number of the participants trained to administer nasal glucagon.

**Table 3:** Practical skills and knowledge and confidence in the methods of administering glucagon.

*Practical skills and knowledge and confidence in the methods of administering glucagon*	Participants trained to use intramuscular glucagon (*n* = 45)	
	*N*	%
Understand how to use glucagon emergency kit	43	95.6
Agree		
Simple to use glucagon emergency kit	36	80.0
Agree		
In case of severe hypoglycemia I could use IM Glucagon	34	75.6
Agree		
Glucagon emergency kit is intimidating and difficult	4	8.9
Agree		
Multi-step reformulation of IM glucagon is a problem	10	22.2
Agree		
The use of a needle to administer IM glucagon is a problem	25	55.6
Agree		

**Table 4:** Practical skills and Knowledge and confidence in the methods of administering glucagon.

*Practical skills and knowledge and confidence in the methods of administering glucagon*	Participants trained to use nasal glucagon (*n* = 9)		*N*	%
Understand how to use Glucagon Nasal Powder	9	100
Agree		
Simple to use Glucagon Nasal Powder	9	100
Agree		
In case of severe hypoglycemia I could use Nasal Glucagon	9	100
Agree		
Glucagon Nasal Powder delivery method is intimidating and difficult	1	11.1
Agree		
The preparation for use of Glucagon Nasal Powder is a problem	1	11.1
Agree		
The method of use of Glucagon Nasal Powder is a problem	1	11.1
Agree		

## 4 Discussion

The aim of the study was to evaluate the effectiveness of the online school staff training programs conducted by healthcare professionals during COVID-19 pandemic. Previously other studies have demonstrated the effectiveness of training with school staff for the management of the child with T1D at school in terms of knowledge and skills (20–24), but to date no one has evaluated the effectiveness of a structured training program in online mode. In these studies, many aspects (theoretical knowledge, self-confidence, and practical skills) were analyzed separately and we decided to validate a new questionnaire that comprehensively included all aspects to better evaluate the effectiveness of the online training program. Telemedicine is an established useful tool for the management of chronic pathologies, and it is very important to evaluate the effectiveness of any new educational program carried out in this modality to evaluate their possible uses and future improvements.

Regarding the study population, 19 schools of our region took part in the survey. This number of schools represents only a sample of the total number of schools to whom training is offered by IRCCS IGG Regional Center for Pediatric Diabetology. Since participation was voluntary, not all 68 schools who participated in the program during the 2-year period also participated in the survey. Furthermore, in the schools that took part in the study, the number of non-participants in the program was higher than the number of participants; we expected this result, because the training is often requested by schools due to the presence of only one child with T1D and only a part of the staff participates in the program. Those who did not attend the training program but did participate in the study were useful in evaluating the effectiveness of the training. Most of the participants in our study were teachers, the professionals which work closer to students with T1D and that spend most of the day with them. Thus, teachers are the most interested in testing their knowledge and management skills of T1D and in improving them by attending training programs. Moreover, most of the participants were not science or biology teachers, reflecting that interest on this issue is showed even by non-expert members of school staff. Most of participants came from schools located in province of Genoa, the provincial capital, probably reflecting a greater willingness to participate in the survey by schools operating in the city context.

The second part of the survey on theoretical knowledge was created using very simple questions with multiple choice (one correct choice, one wrong choice), in order to assess the basic knowledge without going too far into specific details. Data of this section have shown a good global knowledge about T1D among school staff of our region: in almost all of the questions, the percentage of wrong answers was not over 15%. However, for each item, the percentage of wrong answers was higher in Group B, confirming the importance of training attendance in increasing knowledge on T1D. As expected, the differences between the two groups were found in very practical areas such as the management of the insulin bolus and hyperglyacemia, but also in the context of the pathogenesis of T1D, still too often confused with that of T2D even from a population with a high level of education such as teachers. Nearly 100% of the school staff stated that the student with T1D can perform all school activities without limitations, showing that regardless of training participation, there are no perceived limitations in school activities for students with T1D, pointing out the effectiveness of the online training program in improving theoretical knowledge about T1D. This is an interesting result, as the online mode does not always guarantee listener attention and reflects on one hand the interest of the school personnel in being informed and able to manage the patient and on the other hand the effectiveness of the training program.

The third part of survey regarding the level of confidence in handling has highlighted the most evident differences between the two groups: participants that attended the trainings showed a greater self-confidence in handling a student with T1D both at school and in extracurricular activities (like school trips) and they reported feeling more confident in supporting students with T1D both with their daily needs (i.e., insulin administration before meals) and during emergencies (i.e., hypoglycaemic events). These results demonstrate the effectiveness of online training in terms of confidence in student management, which is an even more fundamental aspect compared to theoretical knowledge to guarantee the student a peaceful school life without limitations and to guarantee the teacher peace of mind in supporting the student in all his/her daily needs. Furthermore, Group A (participants in online training program) reported to feel more confident about their theoretical knowledge than their practical management skills; this highlights the importance of adequate training on practical skills, which is certainly more difficult with the online mode.

The last part of the questionnaire showed the great autonomy of the T1D student in managing glucose monitoring by themselves and their need for support from teachers to manage episodes of hypoglycaemia. The school nurse is not available in Italy and since it is not the teacher’s responsibility to deal with the administration of drugs, in most cases the support comes from other figures (parents or private nurses or public services’ nurses). However, it is encouraging that many teachers (24.5%) support children in administering insulin even though it is not their duty, showing once again how important the role of training can be in making teachers feel more confident in managing T1D. Furthermore, data confirmed the extreme ease of use of nasal glucagon, which helped to increase teachers’ self-confidence in the use of this drug in case of severe hypoglycaemia. Anyway, good self-confidence has been reported even by participants trained to use intramuscular glucagon.

### 4.1 Limitation of the study

The results of the study show the usefulness of online training in improving knowledge about T1D and in increasing self-confidence of school staff in handling diabetes, but it is important to highlight some important limits of the study. First of all, the voluntary nature of the study implies two major limitations and bias: the selection of respondents to the questionnaire and the loss of a large number of participants in the program. The higher number of non-respondents could have significantly impacted the results obtained. Furthermore, not knowing the number of total employees and the distribution of respondents within the participating schools, it is not possible to calculate a precise response rate; the response rate could be approximated to 25% based on the average number of training participants and total school employees. However, due to the retrospective and anonymous nature of the study, we thought that the survey could still be an efficient tool for obtaining feedback on the effectiveness of the training. Another limit of our investigation is the rather low statistical power of some comparisons, which could have hampered the interpretation of the related results. In particular, the analysis of the number of correct answers strongly pointed out that the participants in the meeting had better knowledge of T1D, but it was difficult to evaluate which individual aspects had determined this difference. For example, correct answers to the items 3, 6, 7, 8, and 12, even if not statistically significant, were all largely prevalent in the group of participants. The corresponding *post hoc* estimates of statistical power were very poor, ranging between 6 and 49% (data not shown). A prospective study including the greatest possible percentage of participants in the training will be useful in future to better analyze the impact of the training program and, not less important, the satisfaction of the school personnel.

## 5 Conclusion and future perspectives

Our study has confirmed the usefulness of online training in improving knowledge about T1D and, above all, in increasing self-confidence of school staff in handling diabetes-related daily needs and emergencies, thus helping to make school a safer place for T1D students. It is advisable to take advantage of new opportunities given by telemedicine, organizing videoconferences or online meetings and trainings, like we used to do during COVID-19 pandemic. In order to improve school staff practical skills even more, it would be helpful to organize more “practical” trainings, i.e., using models with which participants could practice glucagon administration or simulate real situations. We recommend explaining to school staff how to use nasal glucagon during trainings. In fact, nasal glucagon has probably improved school staff self-confidence and, therefore, the possibility to administer the drug rapidly and effectively, reducing the rate of negative outcomes in case of severe hypoglycaemia, even at school. Finally, our study has demonstrated the absolute importance of school staff trainings in improving the self-confidence of teachers in management of T1D at school and consequently the serenity of students during school hours.

## Data availability statement

The original contributions presented in the study are included in the article/Supplementary material, further inquiries can be directed to the corresponding author.

## Ethics statement

Ethical review and approval was not required for the study on human participants in accordance with the local legislation and institutional requirements. Written informed consent from the participants was not required to participate in this study in accordance with the national legislation and the institutional requirements.

## Author contributions

MB and MSc designed the study and wrote the manuscript. GS and BL researched data and wrote the manuscript. VP researched data. DF wrote the manuscript and designed the tables. MSt reviewed the manuscript and contributed to the discussion. FD wrote the manuscript and designed the figures. FR designed the study. SP performed statistical analyses. Gd’A reviewed the manuscript. NM designed the study and contributed to the discussion. All authors contributed to the article and approved the submitted version.
