# Supplementary material for: Management of Type 1 Diabetes in a school setting: effectiveness of an online training program for school staff
Source: Front Public Health. 2024 Jan 4;11:1228975. doi: 10.3389/fpubh.2023.1228975 (PMC10794362; doi:10.3389/fpubh.2023.1228975)
Supplement: Supplementary file 1 [file Table_1.DOCX]

**Supplementary Table 1**. The questionnaire

**Section 1: Socio-demographical characteristics of participants**

| 1.Indicate your age group | < 30 years | 30-39 years | ≥ 40 years |  |
| --- | --- | --- | --- | --- |
| 2. Indicate the duration of your work experience in school | < 5 years | 5-9 years | 10-19 years | ≥ 20 years |
| 3. Indicate your education level | Professional school diploma | High school diploma | Graduation | Postgraduate higher education |
| 4. Indicate your professional role | Teacher | Teacher assistant | Other staff members | Headmaster |
| 5. Indicate the school level where you work | Kindergarten | Primary school | Middle School | High school |
| 6. If you are a teacher indicate your teaching subject | Science subjects | Other subjects | Not Applicable |  |
| 7. Indicate the province of your school | Genova | Savona | La Spezia | Imperia |
| 8. Do you have Type 1 Diabetes? | Yes | No |  |  |
| 9. Does anyone in your family have Type 1 Diabetes? | Yes | No |  |  |

| **Section 2: Level of theoretical knowledge about T1D**   \|  \| ***Correct answer*** \| ***Wrong answer*** \| \| --- \| --- \| --- \| \| 1. Which are the typical T1D symptoms before its diagnosis? \| Increased thirst and number of urinations \| Weight gain \| \| 2. T1D is usually diagnosed: \| In children and young people \| In adults and elders \| \| 3. The therapy of T1D consists of: \| Subcutaneous insulin injections \| Oral tablets \| \| 4. Paleness, shakiness, sweating, difficulty concentrating and tiredness are typical symptoms of: \| Hypoglycemia (low levels of blood glucose) \| Hyperglycemia (high levels of blood glucose) \| \| 5. T1D is caused by: \| An autoimmune process (reaction of the immune system against its own cells) \| Excessive sugar intake \| \| 6. In case of hypoglycemia you must: \| Administer sugar \| Wait for blood sugar to return to normal spontaneously \| \| 7. In case of hyperglycemia you must: \| Call parents for values > 400 mg/dl for more than two hours \| Administer sugar \| \| 8. Before meals, the child with T1D must: \| Administer insulin and wait for the correct waiting time between insulin administration and meal \| Wait until the blood sugar level is normal before eating \| \| 9. Regarding school activities, the child with T1D: \| Can take part in all school activities as his/her fellows \| Cannot take part in physical education or sports activities \| \| 10. In case of hypoglycemia (blood glucose < 70 mg/dl): \| The child must be helped to act immediately to resolve hypoglycemia \| The child can finish what he/she is doing and deal with it later \| \| 11. In case of hyperglycemia: \| The child can finish what she/he is doing and deal with it later \| The child must be helped to act immediately to resolve the episode \| \| 12. In case of hypoglycemia with loss of consciousness: \| You must administer glucagon and activate the health emergency system \| You must put small amounts of sugar into the child’s mouth \|   **Section 3: Level of self-confidence in handling T1D**   \| 1. How would you rate your knowledge about T1D? \| Poor \| Fair \| Satisfactory \| Good \| Very Good \| \| --- \| --- \| --- \| --- \| --- \| --- \| \| 2. How would you rate your ability to handle T1D? \| Poor \| Fair \| Satisfactory \| Good \| Very Good \| \| 3. How would you rate your ability to recognize symptoms of low blood glucose levels? \| Poor \| Fair \| Satisfactory \| Good \| Very Good \| \| 4. I feel confident handling a child with T1D \| Strongly disagree \| Disagree \| Neutral \| Agree \| Strongly agree \| \| 5. I worry that there are always sugar sachets in the classroom and during school activities in case of low blood glucose levels \| Strongly disagree \| Disagree \| Neutral \| Agree \| Strongly agree \| \| 6. I feel safe supporting the student administering his/her insulin \| Strongly disagree \| Disagree \| Neutral \| Agree \| Strongly agree \| \| 7. I feel confident having a student with T1D in my group during all school activities, including field trips \| Strongly disagree \| Disagree \| Neutral \| Agree \| Strongly agree \| \| 8. I communicate with parents to meet medical needs \| Strongly disagree \| Disagree \| Neutral \| Agree \| Strongly agree \| \| 9. I communicate with healthcare professionals for problems \| Strongly disagree \| Disagree \| Neutral \| Agree \| Strongly agree \| \| 10. How would you rate your experience working with students with T1D? \| Very bad \| Bad \| Satisfactory \| Good \| Very Good \|   **Section 4 - Practical skills and Knowledge and confidence in the methods of administering glucagon**   \| 1.Who helps the child perform glucose monitoring during school hours? \| Him/herself \| Teacher \| Other \| \| --- \| --- \| --- \| --- \| \| 2. Who helps the child manage hypoglycemia during school hours? \| Him/herself \| Teacher \| Other \| \| 3. Who supervises child administer his/ her insulin before meals at school? \| Him/herself \| Teacher \| Other \| \| 4. During the Online Training I was taught to use intramuscular glucagon kit? ***If yes go to Section 4a*** \| Yes \| No \| \| 5. During the Online Training I was taught to use nasal glucagon powder? ***If yes go to Section 4b*** \| Yes \| No \|   **Section 4a – Intramuscular Glucagon Kit**  For each question, please tick the box to indicate your agreement with each statement regarding the device   \| 1a. I understand how to use the Glucagon Emergency Kit \| Strongly disagree \| Disagree \| Neutral \| Agree \| Strongly agree \| \| --- \| --- \| --- \| --- \| --- \| --- \| \| 2a. I think it's simple to use the Glucagon Emergency kit \| Strongly disagree \| Disagree \| Neutral \| Agree \| Strongly agree \| \| 3a. I’m sure that in case of severe hypoglycemia I could use intramuscular glucagon properly \| Strongly disagree \| Disagree \| Neutral \| Agree \| Strongly agree \| \| 4a. The Glucagon Emergency kit delivery method is intimidating and difficult for caregivers like me to use in case of severe hypoglycemia \| Strongly disagree \| Disagree \| Neutral \| Agree \| Strongly agree \| \| 5a. I find that the multi-step reformulation of intramuscular glucagon is a problem \| Not at all \| Somewhat \| Significantly \| Very strongly \| \| 6a. I find that the use of needle to administer intramuscular glucagon is a problem \| Not at all \| Somewhat \| Significantly \| Very strongly \|   **Section 4b – Glucagon Nasal Powder**  For each question, please tick the box to indicate your agreement with each statement regarding the device   \| 1a. I understand how to use the Glucagon Nasal Powder \| Strongly disagree \| Disagree \| Neutral \| Agree \| Strongly agree \| \| --- \| --- \| --- \| --- \| --- \| --- \| \| 2a. I think it's simple to use the Glucagon Nasal Powder \| Strongly disagree \| Disagree \| Neutral \| Agree \| Strongly agree \| \| 3a. I’m sure that in case of severe hypoglycemia I could use nasal glucagon properly \| Strongly disagree \| Disagree \| Neutral \| Agree \| Strongly agree \| \| 4a. The Glucagon Nasal Powder delivery method is intimidating and difficult for caregivers like me to use in case of severe hypoglycemia \| Strongly disagree \| Disagree \| Neutral \| Agree \| Strongly agree \| \| 5a. I find that the preparation for use of glucagon nasal powder is a problem \| Not at all \| Somewhat \| Significantly \| Very strongly \| \| 6a. I find that the method of use of glucagon nasal powder is a problem \| Not at all \| Somewhat \| Significantly \| Very strongly \| |
| --- | --- | --- | --- | --- | --- | --- | --- | --- | --- | --- | --- | --- | --- | --- | --- | --- | --- | --- | --- | --- | --- | --- | --- | --- | --- | --- | --- | --- | --- | --- | --- | --- | --- | --- | --- | --- | --- | --- | --- | --- | --- | --- | --- | --- | --- | --- | --- | --- | --- | --- | --- | --- | --- | --- | --- | --- | --- | --- | --- | --- | --- | --- | --- | --- | --- | --- | --- | --- | --- | --- | --- | --- | --- | --- | --- | --- | --- | --- | --- | --- | --- | --- | --- | --- | --- | --- | --- | --- | --- | --- | --- | --- | --- | --- | --- | --- | --- | --- | --- | --- | --- | --- | --- | --- | --- | --- | --- | --- | --- | --- | --- | --- | --- | --- | --- | --- | --- | --- | --- | --- | --- | --- | --- | --- | --- | --- | --- | --- | --- | --- | --- | --- | --- | --- | --- | --- | --- | --- | --- | --- | --- | --- | --- | --- | --- | --- | --- | --- | --- | --- | --- | --- | --- | --- | --- | --- | --- | --- | --- | --- | --- | --- | --- | --- | --- | --- | --- | --- | --- | --- | --- | --- | --- | --- | --- | --- | --- | --- | --- | --- | --- | --- | --- | --- | --- |
