# Supplementary material for: Management of Type 1 Diabetes in a school setting: effectiveness of an online training program for school staff
Source: Front Public Health. 2024 Jan 4;11:1228975. doi: 10.3389/fpubh.2023.1228975 (PMC10794362; doi:10.3389/fpubh.2023.1228975)
Supplement: Supplementary file 2 [file Table_2.docx]

**Supplementary Table 2**. Section 1: Socio-demographic and work profile characteristics of the study population (N=225)

|  | **N (%)** |
| --- | --- |
| **1) Age group** |  |
| < 30 years | 9 (4) |
| 30-39 years | 32 (14.2) |
| ≥ 40 years | 184 (81.8) |
| **2) Duration of work experience in school** |  |
| < 5 years | 34 (15.1) |
| 5-9 years | 35 (15.6) |
| 10-19 years | 69 (30.7) |
| ≥ 20 years | 87 (38.7) |
| **3) Education** |  |
| Professional school diploma | 5 (2.2) |
| High school diploma | 50 (22.2) |
| Graduation | 126 (56) |
| Postgraduate higher education | 44 (19.6) |
| **4) Professional role** |  |
| Teacher | 179 (79.6) |
| Support teacher | 42 (18.7) |
| Other staff members | 2 (0.9) |
| Headmaster | 2 (0.9) |
| **5) School level** |  |
| Kindergarten | 29 (12.9) |
| Primary school | 68 (30.2) |
| Middle school | 61 (27.1) |
| High school | 67 (29.8) |
| **6) Teaching subject** |  |
| Science subjects | 25 (11.1) |
| Other subjects | 196 (87.1) |
| **7) Province of School** |  |
| Genova | 197 (87.6) |
| Savona | 1 (0.4) |
| La Spezia | 3 (1.3) |
| Imperia | 24 (10.7) |
| **8) Do you have T1D?** |  |
| No | 225 (100) |
| Yes | 0 (0) |
| **9) Does anyone in your family have T1D?** |  |
| No | 207 (92) |
| Yes | 18 (8) |
