# Supplementary material for: Management of Type 1 Diabetes in a school setting: effectiveness of an online training program for school staff
Source: Front Public Health. 2024 Jan 4;11:1228975. doi: 10.3389/fpubh.2023.1228975 (PMC10794362; doi:10.3389/fpubh.2023.1228975)
Supplement: Supplementary file 3 [file Table_3.DOCX]

**Supplementary Table 3.** Section B: Difference in theoretical knowledge about T1D for single item between Group A and Group B.

|  | **Training YES** | **Training NO** | **p-value** |
| --- | --- | --- | --- |
| **1) Which are the typical T1D symptoms before its diagnosis?** |  |  |  |
| Increased thirst and frequent urination | 100% (53) | 96.75% (149) | 0.331 |
| Weight gain | 0% (0) | 3.25 % (5) |  |
| **2) T1D is usually diagnosed in:** |  |  |  |
| Children/ Young people | 98.11% (52) | 90.91% (140) | 0.122 |
| Adult / Elders | 1.89% (1) | 9.09 % (14) |  |
| **3) The therapy of T1D consists of:** |  |  |  |
| Insulin | 100% (53) | 94.81% (146) | 0.117 |
| Tablets | 0% (0) | 5.19% (8) |  |
| **4) Paleness, shakiness, sweating, difficulty concentrating and tiredness are typical symptoms of:** |  |  |  |
| Hypoglycemia | 90.57% (48) | 84.42% (130) | 0.266 |
| Hyperglycemia | 9.43% (5) | 15.58% (24) |  |
| **5) T1D is caused by:** |  |  |  |
| An autoimmune process (reaction of the immune system against its own cells) | 96.23% (51) | 85.06% (131) | **0.031** |
| Excessive sugar intake | 3.77% (2) | 14.94% (23) |  |
| **6) In case of hypoglycemia you must:** |  |  |  |
| Administer sugar | 98.11% (52) | 91.56% (141) | 0.123 |
| Wait for blood sugar to return to normal spontaneously | 1.89% (1) | 8.44% (13) |  |
| **7) In case of hyperglycemia you must:** |  |  |  |
| Call parents only for values > 400 mg/dl for more than two hours | 100% (53) | 93.51% (144) | 0.067 |
| Administer sugar | 0% (0) | 6.49% (10) |  |
| **8) Before meals the child with T1D must:** |  |  |  |
| Administer insulin and wait for the correct waiting time | 94.34% (50) | 83.77% (129) | 0.052 |
| Wait until the blood sugar level is normal before eating | 5.66% (3) | 16.23% (25) |  |
| **9) Regarding school activities, the child with T1D:** |  |  |  |
| Can take part in all school activities as his/her fellow students | 100% (53) | 99.35% (153) | 0.999 |
| Cannot take part in physical education or sports activities | 0% (0) | 0.65% (1) |  |
| **10) In case of hypoglycemia (blood glucose < 70 mg/dl):** |  |  |  |
| The child must be helped to act immediately to resolve the episode | 92.,45% (49) | 92.21% (142) | 0.999 |
| The child can finish what he/she is doing and deal with it later | 7.55% (4) | 7.79% (12) |  |
| **11) In case of hyperglycemia:** |  |  |  |
| The child can finish what he/she is doing and deal with it later | 58.49% (31) | 14.94% (23) | **<0.001** |
| The child must be helped to act immediately to resolve the episode | 41.51% (22) | 85.06% (131) |  |
| **12) In case of hypoglycemia with loss of consciousness:** |  |  |  |
| You must administer glucagon and activate the health emergency system | 98.11% (52) | 91.56% (141) | 0.101 |
| You must put small amounts of sugar into the child’s mouth | 1.89% (1) | 8.44% (13) |  |
